# Compositional and functional differences of the mucosal microbiota along the intestine of healthy individuals

**DOI:** 10.1038/s41598-020-71939-2

**Published:** 2020-09-11

**Authors:** Stefania Vaga, Sunjae Lee, Boyang Ji, Anna Andreasson, Nicholas J. Talley, Lars Agréus, Gholamreza Bidkhori, Petia Kovatcheva-Datchary, Junseok Park, Doheon Lee, Gordon Proctor, Stanislav Dusko Ehrlich, Jens Nielsen, Lars Engstrand, Saeed Shoaie

**Affiliations:** 1 Centre for Host-Microbiome Interactions, Dental Institute, King’s College London, London, UK; 2 Department of Biology and Biological Engineering, Chalmers University of Technology, Gothenburg, Sweden; 3 Stress Research Institute, Stockholm University, Stockholm, Sweden; 4 Department of Psychology, Macquarie University, Macquarie Park, NSW Australia; 5 Department of Medicine Solna, Karolinska Institutet, Stockholm, Sweden; 6 University of Newcastle, Newcastle, NSW Australia; 7 Division of Family Medicine and Primary Care, Department of Neurobiology, Care Sciences and Society, Karolinska Institutet, Stockholm, Sweden; 8 Wallenberg Laboratory, Department of Molecular and Clinical Medicine, University of Gothenburg, 41345 Gothenburg, Sweden; 9 CAS Key Laboratory of Separation Science for Analytical Chemistry, Scientific Research Center for Translational Medicine, Dalian Institute of Chemical Physics, Chinese Academy of Sciences, Dalian, 116023 China; 10 Department of Bio and Brain Engineering, KAIST, 291 Daehak-ro, Yuseong-gu, Daejeon, 34141 Republic of Korea; 11 Metagenopolis, Institut National de la Recherche Agronomique, Jouy en Josas, France; 12 BioInnovation Institute, Ole Maaløes Vej 3, 2200 Copenhagen N, Denmark; 13 Centre for Translational Microbiome Research (CTMR), Department of Microbiology, Tumor and Cell Biology, & Science for Life Laboratory, Karolinska Institute, Stockholm, Sweden; 14 Science for Life Laboratory, KTH-Royal Institute of Technology, Tomtebodavägen 23A, 17165 Solna, Sweden

**Keywords:** Metagenomics, Microbiome

## Abstract

Gut mucosal microbes evolved closest to the host, developing specialized local communities. There is, however, insufficient knowledge of these communities as most studies have employed sequencing technologies to investigate faecal microbiota only. This work used shotgun metagenomics of mucosal biopsies to explore the microbial communities’ compositions of terminal ileum and large intestine in 5 healthy individuals. Functional annotations and genome-scale metabolic modelling of selected species were then employed to identify local functional enrichments. While faecal metagenomics provided a good approximation of the average gut mucosal microbiome composition, mucosal biopsies allowed detecting the subtle variations of local microbial communities. Given their significant enrichment in the mucosal microbiota, we highlight the roles of *Bacteroides* species and describe the antimicrobial resistance biogeography along the intestine. We also detail which species, at which locations, are involved with the tryptophan/indole pathway, whose malfunctioning has been linked to pathologies including inflammatory bowel disease. Our study thus provides invaluable resources for investigating mechanisms connecting gut microbiota and host pathophysiology.

## Introduction

Hundreds of thousands of microbial species colonize the mammalian intestine constituting the gut microbiota^1–3^. This ensemble of species has co-evolved into a complex community in close proximity with the host, developing a symbiosis that provides the host with fundamental functions: protection against pathogens, assimilation of indigestible food, production of essential vitamins, homeostasis maintenance, and immune system development^4,5^.

The gut microbiota composition is determined by host genetics, diet, lifestyle, ethnicity, and living environment, promoting an important inter-individual variability^6^. The most dominant phyla, in a healthy adult human gut, are Bacteroidetes, Firmicutes, and Actinobacteria^7,8^. The distribution of specific families, however, is determined by the above-mentioned factors and by local physiological differences, such as pH, oxygen, and nutrients^7^. The gut microbial community composition can therefore rapidly shift in response to both localised and systemic changes. An altered microbiome composition can lead to dysbiosis, which has been considered to affect the onset and progression of several pathologies, such as inflammatory bowel disease, irritable bowel syndrome, diabetes mellitus, obesity, and colorectal cancer^9–11^.

Biogeography of gut microbes is quite heterogeneous^4,12^. Compared to the gut lumen, the mucus covering the gut mucosa harbours fewer bacteria^7^. The mucus layer lining the epithelium, in particular, is colonised by a unique microbial community, including species such as *Bacteroides fragilis* and *Akkermansia muciniphila*^13,14^. The importance of *A. muciniphila* is only starting to emerge in connection with several diseases: it is most beneficial to the host, although an excess has been linked to pathologies such as multiple sclerosis^15^ and Parkinson’s disease^16^. *B. fragilis* has been mostly studied because of its pathogenicity^17,18^, although it has also been found to provide beneficial effects^19,20^. Having evolved in closer proximity with the host than any other microbe, they affect the host’s health in several ways that still lack a proper understanding. It is therefore of great interest to further investigate them in their own niche^7,21^.

The majority of gut microbiome studies have employed faecal sampling for microbiota screening. To investigate the gut mucosa microbiota, mucosal biopsies, differently from stool samples, would allow the collection of specific microbial communities. The only studies available on colonic mucosal biopsies have employed 16S ribosomal DNA amplicon sequencing (16S)^4,12,22,23^ or RNA sequencing^24^. Although 16S, in particular, has been successfully used to learn about the gut mucosal microbiota composition^25,26^, it has been recently argued that this technique may not be able to reliably resolve taxonomy beyond the genus level, thus precluding a detailed description of locally enriched microbial species^27^.

In this study, we employed shotgun metagenomic sequencing, and a set of functional analysis methods, to investigate the microbiome along the large intestinal mucosa in a healthy subjects’ cohort. The main aim was to investigate if and how this technology can contribute to our understanding of the mucosal microbiota composition and function. We found that, while faecal samples provide a good approximation of the average gut mucosal microbiota, only biopsies could detail subtle but important compositional variations along the intestine. Signature species were identified at each biopsy location, and found, through functional analyses, to affect specific pathways which are known to affect certain physio/pathologic processes.

## Results

**Figure 1:**
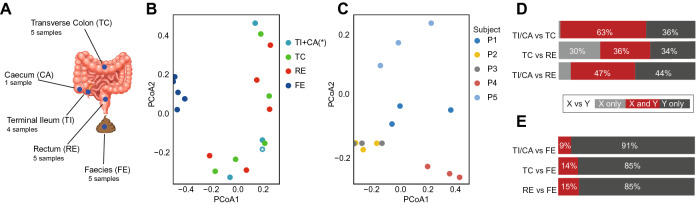
Sampling locations and general overview of the faecal and biopsy-derived metagenomic datasets. (**A**) For each of the 5 enrolled subjects, gut mucosa biopsies were collected from terminal ileum (TI), transverse colon (TC), and rectum (RE); in one subject, as it was not possible to reach her TI, one biopsy was taken from the adjacent caecum instead (CA). Faeces (FE) were also sampled. (**B**, **C**) PCoA plots of the downsized biopsy-faeces dataset (**B**), colour-coded by sampling-location, and of biopsies data only (**C**), colour-coded by subject. The only caecal sample of this study is indicated by an asterisk. (**D**, **E**) Percentage of shared metagenomic species (in dark red) between biopsy location pairs (**D**), and between biopsy locations and FE (**E**).

To investigate the microbial biodiversity along the large intestine mucosa, we collected faeces samples and, after bowel cleansing, mucosal biopsies from 5 healthy Swedish adults chosen from a previously published study^28^: four males and one female, average age 41 years (Supplementary Table 3). Biopsies were taken from three locations: terminal ileum (TI), transverse colon (TC), and rectum (RE). As TI was not accessible for subject P1 (Supplementary Table 3), one of her biopsies was taken from an adjacent caecum (CA) location instead (Fig. 1A). All samples were prepared for shotgun metagenomics sequencing. In the next sections, we detail how these data was used to investigate the microbial compositions of the biopsies and faeces samples (Supplementary Fig. 1).

### Individual microbial uniqueness is stronger than local mucosal microbiota variability

To evaluate the microbial biodiversity in our samples, we first computed their gene- and metagenomic species (MGS)-richness^29^. We downsized biopsy and faeces datasets together to take into account the different sequencing depths across samples (Supplementary Fig. 2A). In the average, biopsies showed an 82,876 gene-richness, and a 23 MGS-richness. Both these values were about one order of magnitude lower than the corresponding ones for faecal samples (1,093,261 and 259, respectively). By merging the samples of all biopsy locations together, we observed a visible increase in their average gene-richness, as certain genes were exclusively observed in specific samples (Supplementary Fig. 2B). Both gene- and MGS-richness showed no significant difference among biopsy locations (Supplementary Fig. 3).

**Figure 2:**
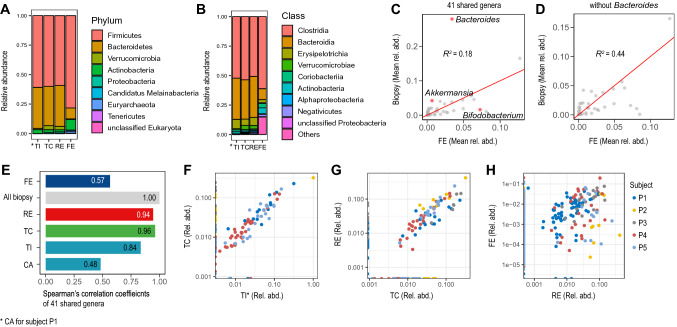
Taxonomy of metagenomics species (MGSs) from terminal ileum/caecum (TI*), transverse colon (TC), rectum (RE), and faeces (FE); n = 5 for each group. (**A**, **B**) Top 10 most highly abundant phyla (**A**) and classes (**B**) in the large intestine; phyla/classes are sorted, in the legends, from the most to the least abundant in all three biopsy-locations. The corresponding faecal values are also plotted for comparison, in the same order. (**C**, **D**) Scatter-plots of mean relative abundances of all 41 genera shared by biopsies and FE (**C**), and of the same genera except *Bacteroides* (**D**). **E** Spearman’s linear correlation coefficients between all biopsies and each sampling location. (**F**–**H**) Scatter-plots of the genera relative abundances of three sample-pairs: TI* versus TC (**F**), TC versus RE (**G**), and RE versus FE (**H**); data points are colour-coded by subject.

**Figure 3:**
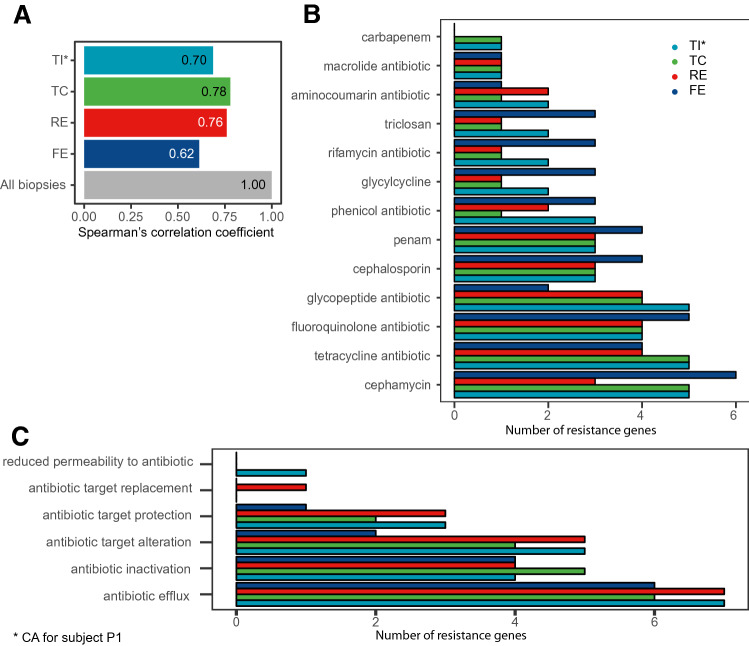
Analysis of antimicrobial resistance genes (ARGs) across biopsy locations (terminal ileum plus caecum (TI*), transverse colon (TC), and rectum (RE)), and in faeces (FE) samples (n = 5 for each group). (**A**) Spearman’s correlation coefficients between ARGs relative abundances of all biopsies and each sampling location. (**B**, **C**) Differential enrichment of drug classes (**B**), or resistance mechanisms (**C**).

We next looked at how subject- and sampling-location factors contributed to the microbiome diversity. The PCoA plot of the faeces-biopsies dataset showed that the faecal microbiome clustered separately from all the biopsy ones (Fig. 1B). While the biopsies microbiomes did not cluster according to their sampling-location (Fig. 1B), they did partially cluster based on subjects (Supplementary Fig. 4A). This shows that faecal and biopsy microbiomes significantly differ from each other, and that this difference is stronger than individual uniqueness. The PCoA plot of biopsies alone showed a clear clustering based on subjects (Fig. 1C), but no clustering based on sampling-location (Supplementary Fig. 4B). This suggests that local microbiomes along the length of the large intestine mucosa have subtle differences, which are overcome by individual variability.

**Figure 4:**
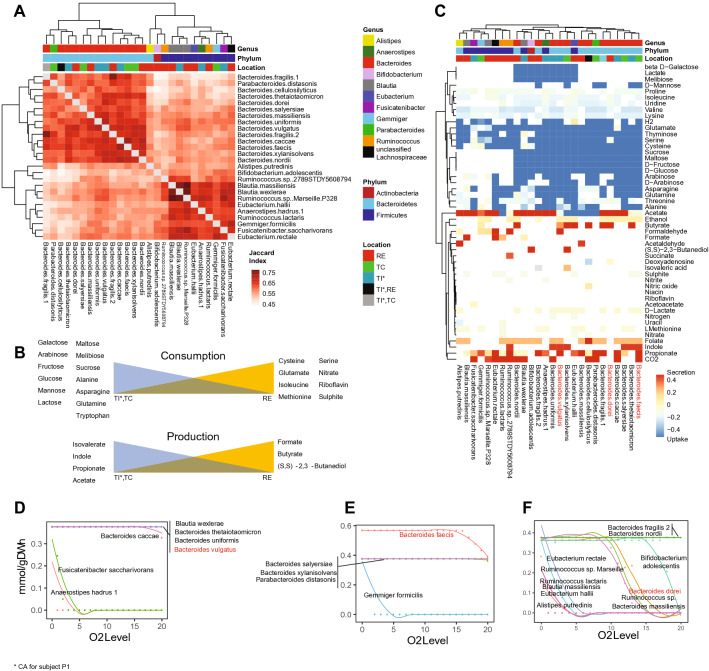
Simulation results of the genome-scale metabolic models of all *Bacteroides* and *Bifidobacterium* species detected in biopsy samples, plus the most highly enriched species at one biopsy location only in at least two patients (Supplementary Fig. 10; Supplementary Table 1). Results are reported by biopsy location: terminal ileum plus caecum (TI*), transverse colon (TC), and rectum (RE). (**A**) Jaccard index for each modelled species. (**B**) Biopsy location-based summation of the secretion of the main bacterial metabolites. (**C**) Uptake and secretion of the metabolites showing a variation across the modelled species. (**D**–**F**) Growth rate plots of the modelled species as a function of environmental oxygen level for TI (**D**), TC (**E**), and RE (**F**). The most enriched *Bacteroides* of the three biopsy locations are highlighted in red (**C**–**F**).

To better interpret these findings, we computed the number of shared MGSs between samples (Fig. 1D,E), and between subjects (Supplementary Fig. 5). We observed a considerable MGS overlap between biopsy locations (Fig. 1D), especially between TI/CA and TC (63%). On the other hand, Fig. 1E shows that, whereas almost all the MGSs detected in biopsies were also detected in faeces (only one MGSs detected in TC, *Acetobacter*, was not detected in faeces), the strong separation between faeces and biopsies (Fig. 1B) was due to the much higher faecal microbial richness. As an additional test, we merged all the data derived from any biopsy location, and we downsized and normalised it together with the faecal dataset. This resulted in a tremendous loss of MGSs for the faecal samples due to the downsizing, which subsequently led to a slight increase in the number of biopsy-only MGSs (Supplementary Fig. 6). Within all the MGSs detected in any biopsy-microbiome, 30% were shared by at least two subjects, (Supplementary Fig. 5), while this percentage, for the faecal microbiome, reaches 54% (Supplementary Fig. 5). This indicates that individual variability is higher in biopsies than in faeces. These results show that, although biodiversity was higher in faeces, since all the MGSs found in biopsies were detected in faeces as well, faecal sampling provides a good approximation of the average gut mucosa microbiota.

### The biopsy-derived gut mucosal microbiomes offer a detailed insight into the biogeography of the large intestine

We next performed a taxonomic analysis of biopsies and faecal microbiomes, in order to assess which MGSs set the two groups apart, and which ones characterize specific biopsy locations. We first computed the total number of MGS-reads belonging to each phylum/class. Figure 2 shows all nine detected phyla (Fig. 2A) and the top-ten classes (Fig. 2B), sorted from the most to the least abundant in biopsies. Phylum Firmicutes was the dominant one, both in faeces and in biopsies, and it was mostly comprised of Clostridia. The phylum Bacteroidetes was the second most represented in biopsies (all belonging to class Bacteroides), whereas it was third in faeces after Actinobacteria (Supplementary Fig. 7). We also performed a similar phylum-enrichment analysis on the dataset previously obtained by merging all biopsies data together. This resulted in a phyla distribution that was in agreement with the one observed for separate biopsy locations (Fig. 2A), once again highlighting Firmicutes as the richest phylum, while the percentage of Bacteroidetes was significantly more abundant in biopsies compared to faeces. (Supplementary Fig. 8). Our findings are in agreement with the current knowledge^7,30^.

To identify the co-variations of faecal and mucosal microbiota across samples, we compared the microbiome of all biopsies as a group to the faeces one. To this purpose, we plotted the 41 genera that were shared by these two groups (Fig. 2C). The correlation between faeces and biopsies was low (R^2^ = 0.18) because of the genus *Bacteroides*, which was significantly enriched in biopsies only (Fig. 2C). The removal of this genus from the shared genera resulted in a significantly higher correlation between biopsies and faeces (R^2^ = 0.44; Fig. 2D). The linear correlation of all shared 41 genera, including *Bacteroides*, between single biopsy locations, was high (R > 0.84), with the exception of CA (R = 0.48) (Fig. 2E–G), whereas RE and faeces had a lower correlation (R = 0.57; Fig. 2H). While these results (Fig. 2E–H) agree with our previous observations, they also show that all the subjects equally contribute to all correlations. We can therefore conclude that the most relevant discrimination factor between faeces and biopsies is genus *Bacteroides*.

Intrigued by the high *Bacteroides* enrichment in all biopsy microbiomes, we looked at which *Bacteroides* species were detected in each biopsy location. Known to be dominant in the gut microbiome^31^, *B. vulgatus* was detected in all samples. Several *Bacteroides* showed specifically high local relative abundances. *B. vulgatus*, *B. thetaiotaomicron*, *B. uniformis*, and *B. caccae* were highest in TI, *B. faecis* in TC, while *B. dorei* and *B. nordii* were only detected in RE. We also observed a decreasing gradient distribution of most of the dominant *Bacteroides* of TI along the large intestine, except for *B. caccae* (Supplementary Fig. 9).

The most enriched genus in the faecal microbiota was the carbohydrate-fermenting genus *Bifidobacterium* (phylum Actinobacteria) (Fig. 2C). *B. adolescentis* and *B. longum* were detected in the faecal samples of almost all subjects (Supplementary Fig. 10). *B. adolescentis*, particularly, displayed an increasing gradient distribution along the large intestine, although its detection in biopsies was significantly lower than in faeces (Supplementary Fig. 10).

Our results support our hypothesis that, except *Bacteroides*, faeces metagenomics provides a good approximation of the average mucosal microbiota. Faecal metagenomics, however, lacks the additional dimension provided by biopsies: a measure of the subtle changes MGSs undergo along the intestine. Since certain pathologies, such as Crohn’s disease, are known to develop at specific locations along the intestine^32–34^, the ability to detect a pathological dysbiosis at such locations is likely to be fundamental for early diagnosis.

### Antimicrobial resistance genes distribution along the large intestine

As the gut microbiota is a reservoir of antimicrobial resistance genes (ARGs)^35^, we next investigated how microbial communities along the intestine contribute to antibiotic resistance. We found that TI was less ARG-rich than both TC and RE, while faeces were significantly richer than any biopsy location (Supplementary Fig. 11). This indicates that TI microbiome may be more susceptible to antimicrobials than those from other locations.

We then looked at how the resistomes of this study correlate with one another (Fig. 3A, Supplementary Fig. 12). The faecal resistome had a low correlation both with all biopsies grouped together (R = 0.62) (Fig. 3A) and with each single biopsy location (Fig. 3C). Biopsies’ resistomes, on the other hand, had higher correlations with each other (R > 0.70). These results show that biopsies, besides having very similar microbiota, had highly overlapping resistomes as well.

We next investigated the differentially enriched drug classes (Fig. 3B) and resistance mechanisms (Fig. 3C). The drug classes with the highest number of ARGs were cephamycin, tetracycline, fluoroquinolone, and glycopeptide antibiotics, particularly in TI and TC (Fig. 3B). The mechanisms with the highest number of ARGs were *Antibiotic efflux, Antibiotic inactivation*, and *Antibiotic target alteration* (Fig. 3C), with particular biopsy locations enrichments. Specifically, *Antibiotic efflux* and *Antibiotic target alteration* were more enriched in TI and RE, while *Antibiotic inactivation* was more enriched in TC. Interestingly, some mechanisms were exclusively represented in one location only, such as *Antibiotic target replacement* in RE, and *Reduced permeability to antibiotic* in TI.

### Functional analysis by KEGG orthology, antiSMASH, and genome-scale metabolic models

To gain a deeper insight into the roles played by locally dominant species along the gut mucosa, we employed a set of functional analysis tools. We first measured how each sample was enriched in specific molecular functional orthologs by assigning KEGG orthologies^36–38^ (https://www.genome.jp/kegg/ko.html) to genes detected at each biopsy location. Two KEGG orthology pathways were found to be significantly enriched (p-value < 0.05) at specific biopsy locations (Supplementary Fig. 13): carotenoid biosynthesis and oxidative phosphorylation. The second was enriched in TI, because of locally dominant bacteria such as *Bacteroides vulgatus* and *Bacteroides uniformis*, while carotenoid biosynthesis was enriched in TC due to *Bacteroidetes vulgatus*, *Akkermansia muciniphila, Faecalibacterium prausnitzii*, *Parabacteroides distasonis*, among others. As carotenoids have been shown to play a protective role in the human gut by regulating immunoglobulin A (IgA) production^39^, we speculate these microbes may play a role not only in carotenoid synthesis but also in regulating and preserving the gut immune system.

The antiSMASH database^40^ was next employed to annotate the faecal and the biopsy-derived data, to predict which secondary metabolites (SMs) are preferentially secreted by the microbes found in a specific sample-type. We found a greater enrichment of such predicted metabolites in the faeces samples, compared to all the biopsy locations, due to the higher number of microbial species detected in faeces (Supplementary Fig. 14A). Within biopsies, TC was the most enriched location for number of identified species with secondary metabolites detected. Nine SMs were predicted to be enriched in all biopsy locations, and an additional five were only detected in TC and RE (Supplementary Fig. 14B). Among these, we found anti-bacterial SMs resorcinol and bacteriocin (secreted by *B. dorei*, *B. faecis*, *B. massiliensis*, and *B. cellulosilyticus*, among others), and aryl polyene (secreted by *Akkermansia muciniphila*, and by most *Bacteroides* species), which provides bacteria with protection from oxidative stress similarly to carotenoids^41^.

We then employed genome-scale metabolic modelling (GEM) to investigate how locally-enriched microbes contribute to the metabolism of the gut microbiota and the host. Specifically, we constructed and simulated GEMs of all the *Bacteroides* detected in biopsies, and of all the species that were most highly enriched at a single biopsy location, in at least two subjects (Supplementary Fig. 15).

The Jaccard similarity analysis between the simulation results of all modelled species was in agreement with our former observations: there was no marked difference between biopsy locations (Fig. 4A). However, several metabolites were especially, although not exclusively, predicted to be produced/secreted at certain biopsy locations because of locally-enriched species (Fig. 4B,C). In particular, we report a gradual shift from sugar to amino acid consuming microbes (Fig. 4B). As expected^42^, acetate secretion (highest in TI) was predicted to be greater than propionate’s, which was greater than butyrate’s (Supplementary Table 2). Formate and butyrate production was highest in RE (Fig. 4B), where *Ruminococcus lactaris*, *Eubacterium rectale*, and *B. nordii* were enriched (Fig. 4C), while propionate production was highest in TI (Fig. 4B), which had higher concentrations of *B. caccae*, *B. vulgatus*, and *B. fragilis* (Fig. 4C).

Our simulations yielded novel interesting insights into several metabolic pathways whose relevance to health or disease has already been assessed, but whose mechanisms are still largely obscure. In particular, we predicted significantly enriched indole secretion and l-tryptophan (Trp) consumption in TI and TC (Fig. 4C), mainly due to *B. thetaiotaomicron*, *B. uniformis*, *B. vulgatus*, *B. faecis*, and *B. xylanisolvens*. Trp is an essential amino acid involved in several important functions, mostly connected with the host-microbiome interaction. As a fraction of Trp is known to be metabolised into indole by the gut microbiota^43^, our results show that this process is likely to happen in TI and TC (Fig. 4C).

Finally, by simulating the growth of the modelled species with varying oxygen concentrations, we predicted different responses from locally dominant *Bacteroides*. In particular, *B. vulgatus* (dominant in TI) was predicted to have a high oxygen-resistance (Fig. 4D), whereas *B. faecis* (dominant in TC) is likely to be more sensitive (Fig. 4E), and *B. dorei* (dominant in RE) could be quite oxygen-sensitive (Fig. 4F).

## Discussion

The current ideal strategy to study gut mucosal microbial communities is to sample them through biopsies and to employ a shotgun sequencing technology to measure their microbial species composition. A number of factors, however, need to be taken into account when choosing the most practical strategy for each specific study, including sample size, sequencing costs, and desired sequencing depth. This study was designed to explore the advantages and limitations of using shotgun metagenomics and gut mucosal biopsy samples to quantify local microbiomes along the intestine of healthy subjects.

We show that faecal samples are richer in biodiversity. This is due, at least partially, to the expected lower number of reads mapping to microbial genes in biopsy-derived samples, as these contain higher amounts of human DNA. We also found important individual differences, particularly in the biopsy microbiota, less so in the faecal ones. These observations are in agreement with the findings of Zoetendal et al., who compared faecal and mucosal biopsy samples of human subjects by 16S, highlighting significant differences between the mucosal microbiota compositions of different individuals^44^. Biodiversity was, therefore, the main discriminating factor between faeces and biopsies microbiota, followed by individual variability, while biopsy sampling-location offered the least discriminating power across biopsies. Although not significantly different in composition, different gut locations vary in species concentrations: the same species may be found in most or all the biopsy locations, but with a different prevalence within each local community. Such variations are of crucial interest, as they may affect the host metabolism and homeostasis, or the onset and development of certain diseases where an altered composition of local communities is linked with such processes.

Despite the richer faecal biodiversity, faecal microbiota included almost all the MGSs found in mucosal biopsies. Additionally, faecal and biopsy microbiomes correlate with each other very well, except *Bacteroides*. This suggests that faeces metagenomics provides a good approximation of the average gut mucosal microbiota composition, in health. Regarding sequencing depth, we applied a downsizing (rarefaction) approach to avoid possible biases. There are, however, reports in the literature describing the low impact of downsizing on read-counts of shotgun metagenomics, as this method does not significantly confound biological effects^45^. Our downsizing approach reduced the number of species identified, but it allows for an unbiased understanding of the faecal and mucosal microbiome.

Both faeces and biopsies were particularly rich in Firmicutes, a phylum known to be dominant in the gut microbiota^7^. Several microbial species belonging to genus *Bacteroides* were, however, significantly enriched in biopsies only, whereas species from genus *Bifidobacterium* were mainly enriched in faeces. These results are in agreement with the phyla distribution described by 16S studies^4^. *Bacteroides* are known as obligate anaerobic, bile-resistant bacteria, and one of the dominant genera in the gut^46^. Although they often behave as commensal organisms, some of their features allow them to turn into pathogens^46,47^. *Bacteroides* species have been recently discovered to enhance gut homeostasis by secreting immunomodulatory factors^48^. *B. fragilis*, in particular, is the most studied species of this phylum. It has been shown to play an anti-inflammatory role, to promote mucosal colonization, and to enforce the epithelial barrier of the gut^49–51^. There is thus increasing interest in *Bacteroides* species for their potential in the treatment of a number of diseases. They are among the rare prokaryotes provided with membrane sphingolipids, which are believed to enhance their ability to detect and cope with an unstable environment^52^. Furthermore, *Bacteroides*’ outer membrane comprises lipopolysaccharides^53^; although they usually trigger the host immune response, *Bacteroides* species-specific lipopolysaccharides structural differences prevent that^54^. Overall, *Bacteroides* are equipped with a unique interface with the host. Besides providing them with the capability to quickly detect and react to environmental changes, it also allows them to affect and modulate the host immune system^54^. Moreover, faecal microbiota transplantation has been shown to significantly increase the abundance of *Bacteroidetes* in the gut mucosa^55^. The enrichment of *Bacteroidetes* in the gut mucosa is therefore likely due to a number of niche selection factors that allow this phylum to thrive in this environment even after perturbations, such as the bowels cleansing all our individuals were subjected to before colonoscopy. Among the most enriched *Bacteroides*, *w*e showed *B. thetaiotaomicron* and *B. fragilis* to gradually decrease from TI to RE, while *B. faecis* was significantly enriched in TC. Being among the best-studied *Bacteroides*, they are known as flexible foragers, capable of adapting to changes in microbial composition and glycan availability^56,57^. Their enrichment may, therefore, have been caused by the depletion of other genera caused by bowel cleansing.

We next performed a set of analysis on functional capacity to investigate the roles possibly played by locally dominant species. First, we explored the local enrichment of antibiotic resistance genes. The higher ARG-richness found in faeces was due to the greater MGS-richness of these samples. Interestingly, the number of resistance genes corresponding to several resistance mechanisms was higher in some biopsy locations, particularly TI and RE, than in faecal samples. In particular, although TI had the lowest gene/MGS-richness, and no unique MGSs of its own, it had the highest number of significantly changing ARGs. Some ARGs may exist as a result of acquired resistance to antibiotic use. Antibiotic resistance is known to have significant variations between countries^58^. The most commonly prescribed antibiotics in Sweden, where the individuals of this study are from, are mainly penicillins, classified as penam, followed by tetracycline and fluoroquinolone antibiotics (https://resistancemap.cddep.org/index.php). In our study, the highest number of ARGs was detected for cephamycin, tetracycline, and fluoroquinolone antibiotics, whereas less ARGs were detected for penams. Furthermore, although a few ARGs were shared among more than one drug class, many of them were specific to other classes that, to our knowledge, were not commonly prescribed in Sweden in the past twenty years. Some of these, like ARGs conferring resistance to cephamycin and glycopeptide antibiotics, were quite prominent in our results, and could be derived either from intrinsic resistance or from acquired resistance via horizontal gene transfer^59,60^.

We next performed a KEGG orthology analysis, which resulted in two pathways being significantly enriched, at two separate locations: carotenoid biosynthesis in TC, and oxidative phosphorylation in TI. Our analysis also allowed the identification of the microbial species that most significantly contributed to these local enrichments. Not enough information is however currently available to draw any sound conclusions relative to the oxidative phosphorylation enrichment in TI. Additionally, since our GEMs were simulated in anaerobic conditions, our simulation could not be used to shed light on this enrichment either. Carotenoid biosynthesis, on the other hand, was enriched in TC because of the local prevalence of a few microbes, including *Bacteroidetes vulgatus* and *Akkermansia muciniphila*. The carotenoid biosynthesis pathway is responsible for producing beta-carotene, a precursor for retinol metabolism, and vitamin A. It has been shown that carotenoids play a protective role on the human gut by regulating IgA production^39^, and that retinol deficiency specifically affects *Bacteroides vulgatus*’ growth in the gut^61^. Our data allows us to speculate that microbes possessing genes from this pathway, and locally enriched in TC, may play an important role in the retinol turnover of the gut, and in regulating IgA production.

GEM modelling was used to investigate the secretion/uptake of fermentation metabolites by the most enriched microbial species at each biopsy location. The simulations of our models provided new insights into partially known metabolic mechanisms involving microbial species which were found, in this work, to be dominant at specific locations only. We observed, for instance, a gradual shift, along the intestine, from sugar to amino acid consuming microbes, and we reported unprecedented oxygen-resistance details of specific *Bacteroides* species. Of particular interest was the discovery that Trp consumption and indole production were both significantly increased in TI and TC. Trp is an essential amino acid that plays fundamental roles in regulating nitrogen balance, gut immune system homeostasis, and serotonin production^62,63^. A fraction of Trp is metabolised to indole and its derivatives by the gut microbiota, consistently with our simulation results^62^. Several diseases have been linked with reduced efficiency of this mechanism^63,64^. In particular, inflammatory bowel disease has been associated with decreased serotonin production and Trp imbalance^65^. *Bacteroides* are among the few known microbes possessing Trp metabolism catalytic enzymes^63^. The main *Bacteroides* found in TI and TC that, according to our GEM simulation, were responsible for Trp consumption and indole secretion, were *B. thetaiotaomicron*, *B. uniformis*, *B. vulgatus*, *B. faecis*, and *B. xylanisolvens*. This novel information is extremely valuable, particularly as the impact of this mechanism is only starting to be uncovered in connection with a growing number of diseases. The *Bacteroides* species we report here in connection with Trp/indole metabolism will thus require further targeted investigation, as that could help prevent and treat such pathologies. As the results derived from our GEM simulation were obtained in silico, further targeted experiments will be required to validate them.

One of the main limitations of this study was the small number of subjects that could be enrolled. Very strict inclusion criteria were applied to ensure that all the included subjects were healthy, thus without any organic findings or gastrointestinal symptoms at the time of enrolment, which could have influenced the microbiota composition. Also, the sequencing depth of the biopsy-derived samples (0.15 million unique read-counts) was limited by the large number of human genes sampled together with the microbes. The lower sequencing depth led to a partial measurement of the gut mucosal microbiota, which mainly emphasised the most abundant species. This is another important limitation of metagenomics. A number of studies investigated the composition of the gut mucosal microbiota by 16S instead. This technology allowed them to detect larger numbers of microbial species, including rare ones than our study could. The enormous potential of metagenomics was hampered by the extremely high host genome content in combination with insufficient sequencing depth. Additionally, the bowels cleansing procedure that was required prior to colonoscopy is likely to have caused substantial alterations to the gut mucosal microbiota^66^. As for the coverage of ARGs from the catalogue used here, we recently discovered a new study suggesting a larger set of putative antibiotic resistant genes identified by a deep learning approach^67^. The additional use of this new set may help better understand the differences in ARG-richness in a more comprehensive manner. In addition, a deeper sequencing may lead to the identification of more ARGs from both faecal and mucosal samples. Additionally, normalizing for microbiota richness may provide better understandings of the differences of antibiotic resistance capacity between faecal and mucosal microbiota. While these are important and expected limitations, we still chose to use this method to assess what can be learnt from it. Biopsies can provide information that faeces cannot: subtle and otherwise undetectable variations in local microbiomes along the intestine that faeces cannot detect. Such variations are of great interest since, as discussed, they can affect the onset and progression of serious pathologies. We thus believe these results, although still not optimal, provide new valuable insights into the mucosal gut microbiota. New methods will need to be devised to better isolate microbial species from human tissues, thus increasing the detection of microbial species. In order to achieve results that are at least comparable to those of 16S, a much higher sequencing depth must also be obtained by, for instance, multiple resequencing of the same samples. Additionally, given the promising results here described, larger cohorts now need to be investigated.

In conclusion, the current study showed that faecal samples provide a good approximation of the gut mucosal microbiome, although only metagenomics of gut mucosal biopsies can detect subtle variation in the local microbial communities’ composition along the large intestine. Functional analysis of the biopsy metagenomics data was in agreement with the current knowledge while providing new fundamental information. Our GEM simulation, in particular, could detail which species are involved in only partially known metabolic mechanisms connected with health or disease. Our work provides novel insights into which microbial species are associated with the gut mucosal microbiota of healthy individuals after bowels cleansing. Such valuable information will provide the starting point for more targeted future investigations on the gut microbiota.

## Materials and methods

### Study population

Five healthy adult volunteers from Stockholm were selected from the participants to a study previously described^28^. Colonoscopy preparation included a clear liquid diet and bowel cleansing with 45 mL Phosphoral oral intake twice within 4 h. At colonoscopy, biopsies were collected from terminal ileum or caecum, transverse colon, and rectum. Faecal samples were collected at home, before bowel cleansing and colonoscopy, and sent by post to the research facility where they were frozen at − 80 °C. All subjects were free from any objective finding at colonoscopy. They had not undergone any previous gastrointestinal surgeries and had no current or previous diseases of the gastrointestinal tract (for exclusion criteria, see Supplementary Materials). The study was approved by the Karolinska Institutet ethical review board (Forskningskommitté Syd, nr 394/01). All participants provided written informed consent.

### DNA extraction

Total genomic DNA was isolated from biopsy tissue or from 100 to 120 mg faecal sample using repeated bead beating, following a protocol previously described^68^. Briefly, samples were placed in Lysing Matrix E tubes (MP Biomedicals), and sterile lysis buffer (4% w/v SDS; 500 mmol/L NaCl; 50 mmol/L EDTA; 50 mmol/L Tris·HCl; pH 8) was added. Biopsy samples were incubated with 50 µl mix of mutanolysin (5U/µL) and lysozyme (100 mg/mL) at 37 °C for 30 min. Both biopsy and faeces samples were lysed twice with bead beating at 5.0 m/s for 60 s in a FastPrep-24 Instrument (MP Biomedicals). After each bead-beating, samples were heated at 85 °C for 15 min and centrifuged at full speed for 5 min at 4 °C. Supernatants from the two lysate-fractions were pooled and purified. Total genomic DNA was eluted in AE buffer (10 mmol/L Tris·Cl; 0.5 mmol/L EDTA; pH 9.0). All the experimental protocols employed in this study were in accordance with the relevant guidelines and regulations.

### Analysis of shotgun metagenomics

Extracted DNA was processed into a paired-end library and sequenced by Illumina HiSeq 2,500 (2 × 100 bp), generating an average of 5.9 million paired-end reads per sample. Whereas faecal samples yielded an average of 5.8 million unique read-counts mapping to the integrated reference catalogue of the human gut microbiome database, only an average of 0.15 million unique read-counts mapped to microbial genes in the biopsy-derived samples. This was due to the majority of reads, in biopsy-derived samples, mapping to human genes (97%). These data were thus used to generate two separately normalised gene-count tables and, correspondingly, MGSs abundance profiles (Supplementary Table 4): one for faeces and one for biopsies. Raw data was quality checked with FastQC (https://www.bioinformatics.babraham.ac.uk/projects/fastqc), processed with METEOR^69^, and mapped onto the integrated reference catalogue of the human gut microbiome^70^. Host DNA was removed. Reads that were aligned to the gene catalogue by bowtie2 were counted when both paired-end reads were aligned to given genes. Gene abundances were estimated from the summation of uniquely mapped read counts and normalized shared read counts by occurrences. Estimated gene abundance from uniquely and shared mapped reads were normalized by the length of given genes by MetaOMineR R package^29^.

Downsizing was performed in order to take into account the different sequencing depths across samples. Strain/species-level abundances of MGS—i.e., co-abundant genes with more than 100 genes originated from microbial species^71,72^—were profiled for each sample. Downsizing was performed for the whole biopsy-faeces dataset, then also for biopsy and faeces datasets separately. MGS profiles were estimated as the mean abundance of the 50 genes of a given MGS (centroid of the clustered genes) and used to perform taxonomic investigations. Based on the orthologous genes from the KEGG database^73^, we annotated the quantified bacterial genes, and performed functional analysis of the genes detected in the biopsy dataset.

### Statistical analysis

To assess if and how much each set of data (either sampling location- or patient-specific) differ, in the average, from the others, paired difference tests were performed. In particular, as the available data was not large and did not appear to be normally distributed, statistical assessments were performed with the Wilcoxon signed-rank test^74^, in MatLab.

### Antibiotic resistance genes

All fastq files were mapped against the nucleotide_fasta_protein_homolog_model from the antimicrobial resistance database CARD3.0.0^75^ (https://card.mcmaster.ca/) using Bowtie2 (ver.2.3.4.3). Unmapped reads were filtered out, and resistance genes with mapped reads coverage below 90% were discarded. Each resistance gene was annotated with Drug Class and Resistance Mechanism using CARD3.0.0 metadata. R package Deseq2 was used to normalize the resistome dataset, and to perform statistical evaluation of significantly changing resistance genes.

### Secondary metabolite prediction via antiSMASH pipeline

All the gene sequences of the 606 metagenome species we identified were retrieved from the reference gene catalogue^70^, and the antiSMASH standalone program was used to annotate their biosynthetic genes by minimal run options focused on core detection modules (version 5)^76^. The antiSMASH program was loaded onto the Amazon cloud computing platform (AWS) as docker image, and its mining process was executed per metagenomics species with all processes massively parallelized. All detected secondary metabolite clusters per metagenomics species were then associated with the sampling locations of each metagenomics sample.

### Genome scale metabolic model (GEM) reconstruction and simulation

We reconstructed the GEMs of the bacteria (Supplementary Table 1) belonging to genera *Parabacteroides*, *Anaerostipes*, *Bacteroides*, *Bifidobacterium*, *Eubacterium*, *Ruminococcus*, and *Blautia*, using the KO annotation provided in the gut catalogue. The KO profiles were converted to metabolic network and reaction score profiles regarding the KBase reference model. To make the functional models regarding the provided biomass objective function, the gap filling was done using the raven toolbox. All models were constrained by the general UK diet (https://fdnc.quadram.ac.uk/). Simulations were run anaerobically to calculate the growth rate for each model, and the production profiles of the bacteria. The resulting biomass figures were proportioned by species abundances based on colon locations. We added an initial source of acetate and lactate based on the average production profile of each microbe. For the second simulation, models were constrained both by the predicted biomass of the bacteria and by the diet. The flux balance analysis result (Supplementary Table 2) was used to find metabolite importance for each part of the colon. The sensitivity of the models to oxygen was simulated regarding the oxygen-uptake of the intestine (2 mL O_2_/min × 100 g tissue), where the micro-aerobic condition was considered as 5% O_2_-uptake. All references relative to the GEM method here described are available in Supplementary Materials.

### Graphs generation and figures formatting

All graphs were generated with R, version R-3.5.2 (https://www.r-project.org/). Figures were then created and formatted with Adobe Illustrator 2019 (https://www.adobe.com).

## Supplementary information

Supplementary Information.

Supplementary Table 1.

Supplementary Table 2.

Supplementary Table 4.

## Data Availability

All raw metagenomic data have been deposited in the public EBI/NCBI Database under accession number PRJEB33194.
