# Supplementary material for: Compositional and functional differences of the mucosal microbiota along the intestine of healthy individuals
Source: Sci Rep. 2020 Sep 11;10:14977. doi: 10.1038/s41598-020-71939-2 (PMC7486370; doi:10.1038/s41598-020-71939-2)
Supplement: Supplementary file 1 — Supplementary Information. [file 41598_2020_71939_MOESM1_ESM.pdf]

Compositional and functional differences of the mucosal microbiota along the intestine of healthy individuals

Stefania Vaga[1,§], Sunjae Lee[1, §], Boyang Ji[2], Anna Andreasson[3,4,5], Nicholas J Talley[6], Lars Agréus[7], Gholamreza Bidkhori[1], Petia Kovatcheva-Datchary[8,9], Junseok Park[10], Doheon Lee[10], Gordon Proctor[1], Stanislav Dusko Ehrlich[11], Jens Nielsen[2,12,*], Lars Engstrand[13,*] & Saeed Shoaie[1,14,*]

[1] Centre for Host-Microbiome Interactions, Dental Institute, King's College London, London, UK

[2] Department of Biology and Biological Engineering, Chalmers University of Technology, Gothenburg, Sweden

[3] Stress Research Institute, Stockholm University, Stockholm, Sweden

[4] Department of Psychology, Macquarie University, NSW Australia

[5] Department of Medicine Solna, Karolinska Institutet, Stockholm, Sweden

[6] University of Newcastle, Newcastle, New South Wales, Australia

[7] Division of Family Medicine and Primary Care, Department of Neurobiology, Care Sciences and Society, Karolinska Institutet, Stockholm, Sweden

[8] Wallenberg Laboratory, Department of Molecular and Clinical Medicine, University of Gothenburg, Gothenburg, 41345, Sweden

[9] CAS Key Laboratory of Separation Science for Analytical Chemistry, Scientific Research Center for Translational Medicine, Dalian Institute of Chemical Physics, Chinese Academy of Sciences, Dalian, 116023 China

[10] Department of Bio and Brain Engineering, KAIST, 291 Daehak-ro, Yuseong-gu, Daejeon 34141, Republic of Korea

[11] Metagenopolis, Institut National de la Recherche Agronomique, Jouy en Josas, France

[12] BioInnovation Institute, Ole Maaløes Vej 3, DK2200 Copenhagen N, Denmark

[13] Centre for Translational Microbiome Research (CTMR), Department of Microbiology, Tumor and Cell biology, & Science for Life Laboratory, Karolinska Institute, Stockholm, Sweden

[14] Science for Life Laboratory, KTH-Royal Institute of Technology, Tomtebodavägen 23A, Solna, Sweden, SE- 17165

Supplementary Materials

Study population exclusion criteria:

- Organic findings at the endoscopy
- Previous GI surgery
- Previous or current GI disease or consultations due to GI symptoms
- Known lactose intolerance
- Current use of medicines or current diagnoses
- Antibiotic use previous 6 months
- Smoking
- Snuff use
- Self-reports of abdominal pain, diarrhoea or constipation on the ASQ or Rome II questionnaire
- Overweight
- High blood pressure
- Aberrant blood test (including C-reactive protein and glucose)

Study references for Genome Scale metabolic Modelling:

Arkin AP, Cottingham RW, Henry CS, et al. KBase: The United States Department of Energy Systems Biology Knowledgebase. Nat Biotechnol 2018;36:566–569.

Agren R, Liu L, Shoaie S, et al. The RAVEN Toolbox and Its Use for Generating a Genome-scale Metabolic Model for Penicillium chrysogenum. PLoS Comput Biol 2013;9.

Bidkhori G, Benfeitas R, Klevstig M, et al. Metabolic network-based stratification of hepatocellular carcinoma reveals three distinct tumor subtypes. Proc Natl Acad Sci 2018;115:E11874–E11883.

Lutz J, Henrich H, Bauereisen E. Oxygen supply and uptake in the liver and the intestine. Pflügers Arch Eur J Physiol 1975;360:7–15.

Sigalevich P, Cohen Y. Oxygen-dependent growth of the sulfate-reducing bacterium Desulfovibrio oxyclinae in coculture with Marinobacter sp. strain MB in an aerated sulfate-depleted chemostat. Appl Environ Microbiol 2000;66:5019–5023.

Supplementary Table 1 – Microbial species used for genome scale metabolic modelling. The table file is a separate supplementary file.

Supplementary Table 2 – Genome scale metabolic model information and flux balance analysis results. The table file is a separate supplementary file.

Supplementary Table 3 – Study cohort.

Patient ID	Age	Sex
**P1**	34	F
**P2**	64	M
**P3**	35	M
**P4**	31	M
**P5**	42	M

Supplementary Table 4 – List of all the MGSs that were detected in our samples. The table file is a separate supplementary file.

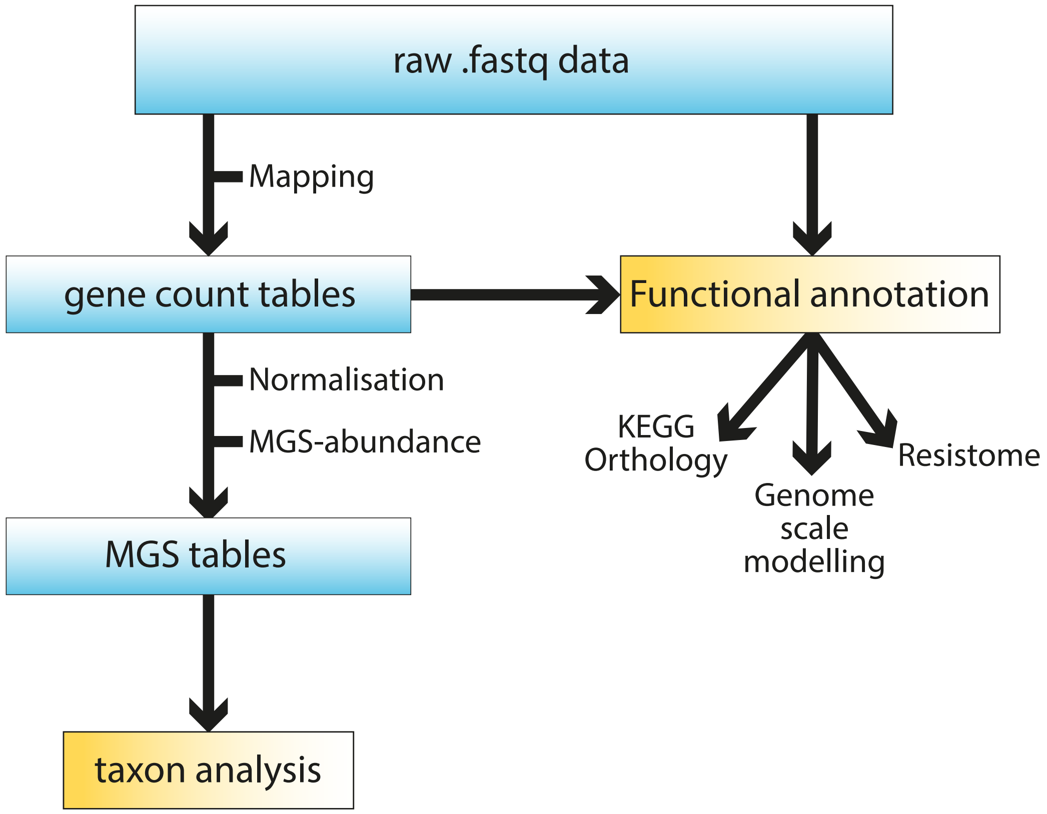

Supplementary Figure 1 - Data analysis workflow for taxonomic and functional investigation of the biopsy/faeces metagenomics data.

A

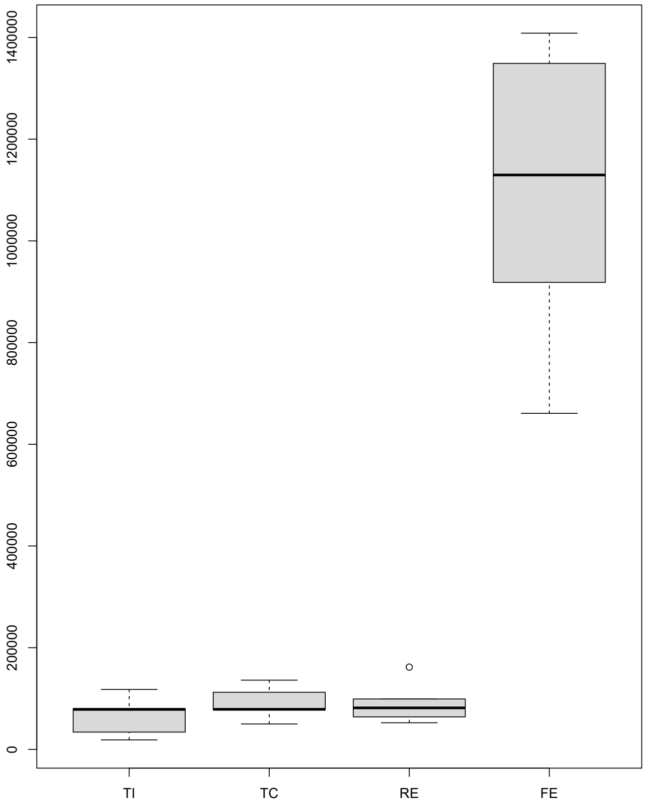

B

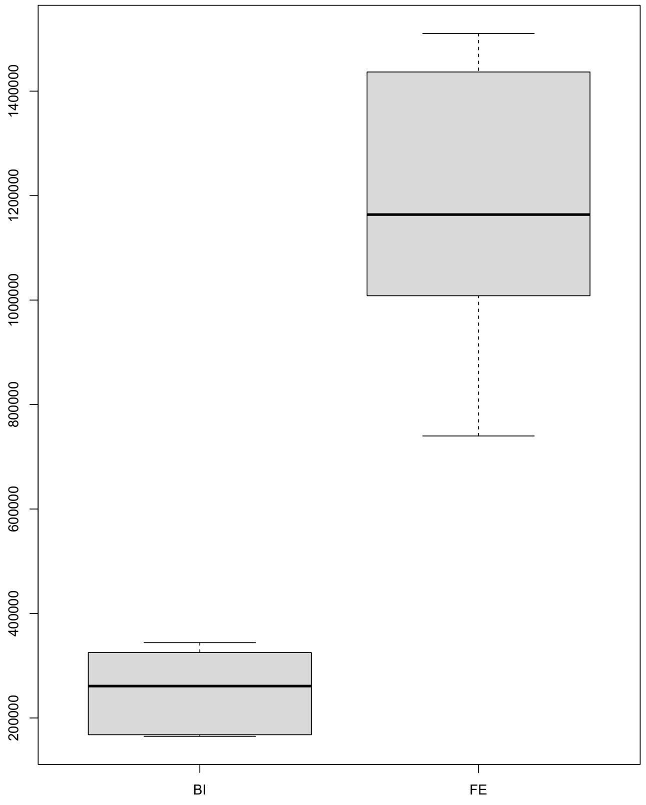

Supplementary Figure 2 - Total number of detected genes (gene richness) (A) for each biopsy location, and (B) for all biopies merged together, compared to faeces.

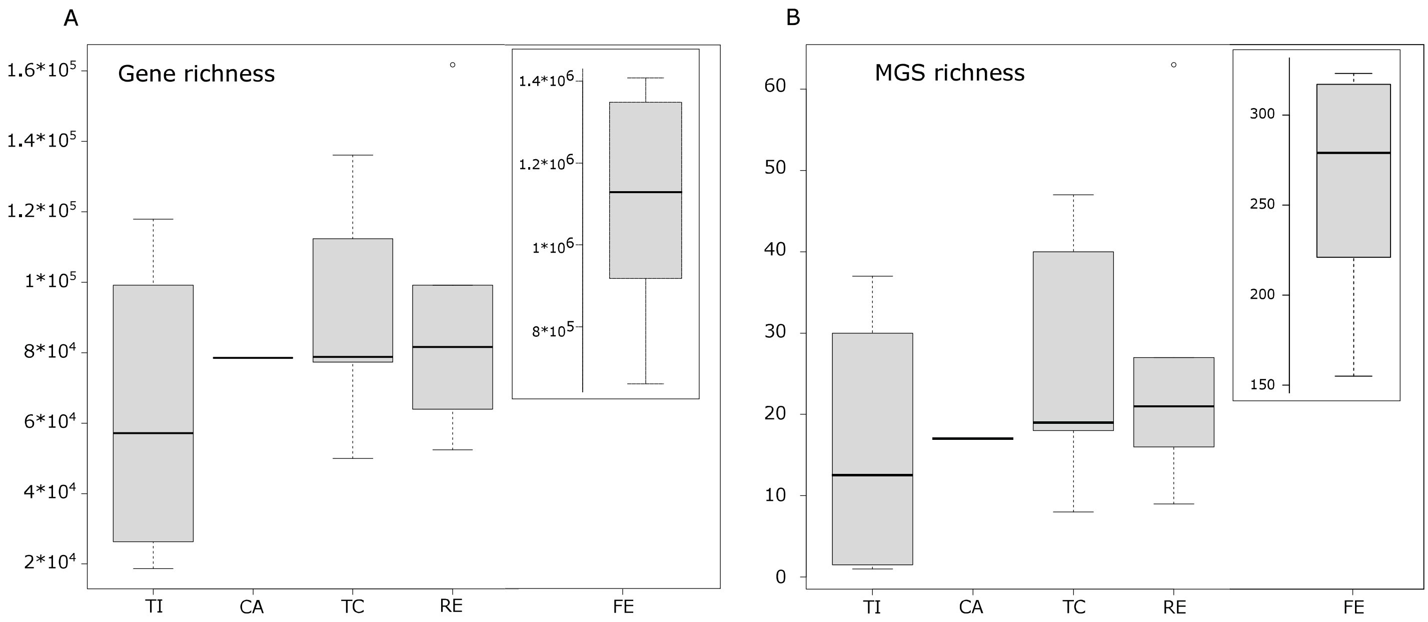

Supplementary Figure 3 - Gene- (A) and MGS- (B) richness boxplots.

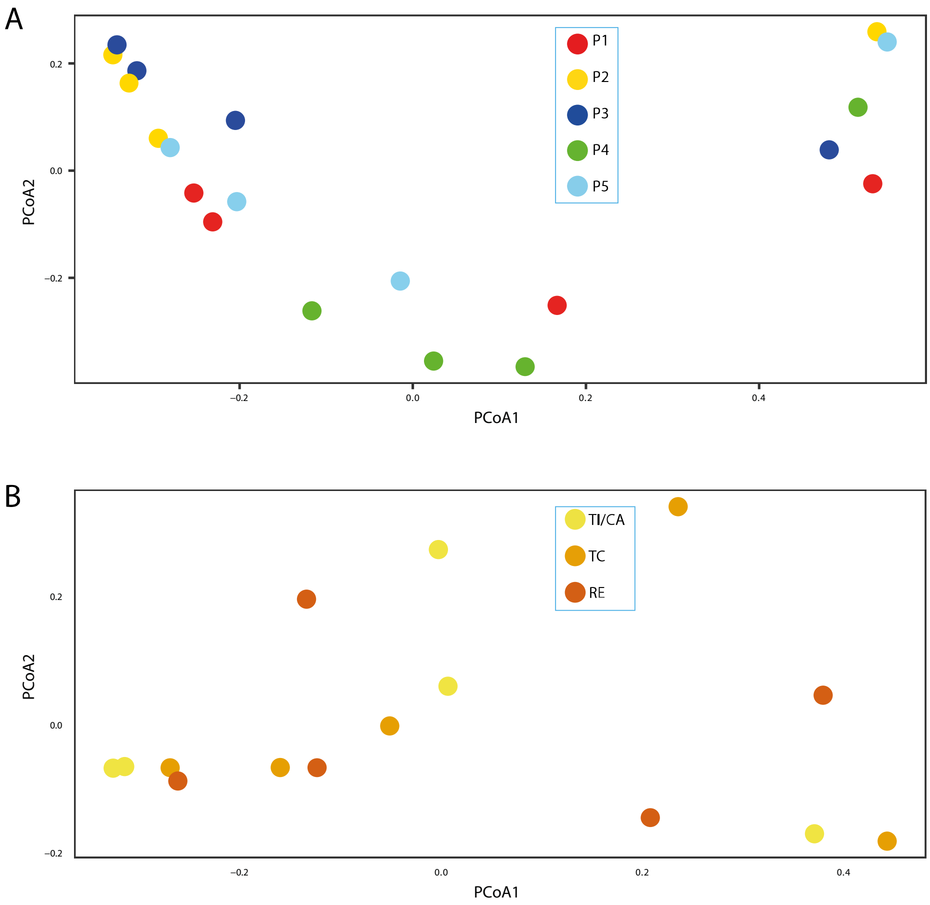

Supplementary Figure 4 - PCoA plots of the complete faeces/biopsies dataset colour-coded for subject (A), and of the biopsies dataset only colour-coded for biopsy sampling location (B).

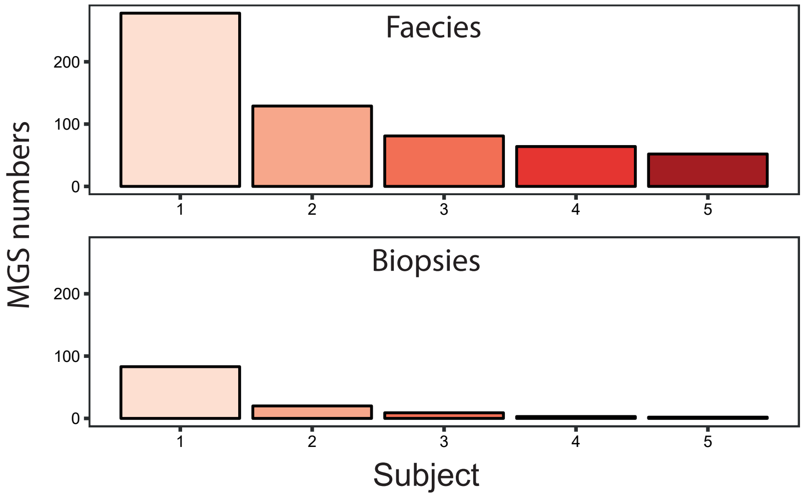

Supplementary Figure 5 - Number of metagenomics species (MGSs) shared between subjects in the faeces dataset (top panel) or in the biopsies one (bottom panel).

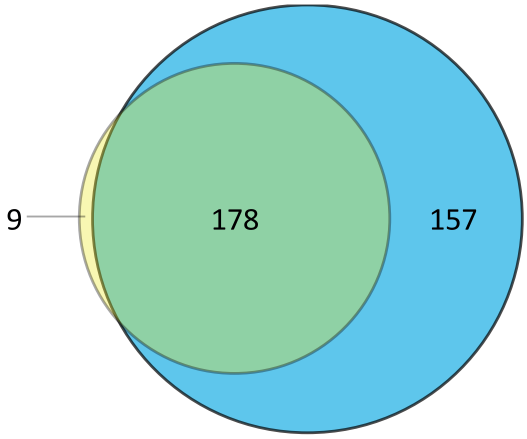

Supplementary Figure 6 - Metagenomics species numbers after merging of all biopsy-derived data, and subsequent downsizing of biopsy and faecal -derived samples together.

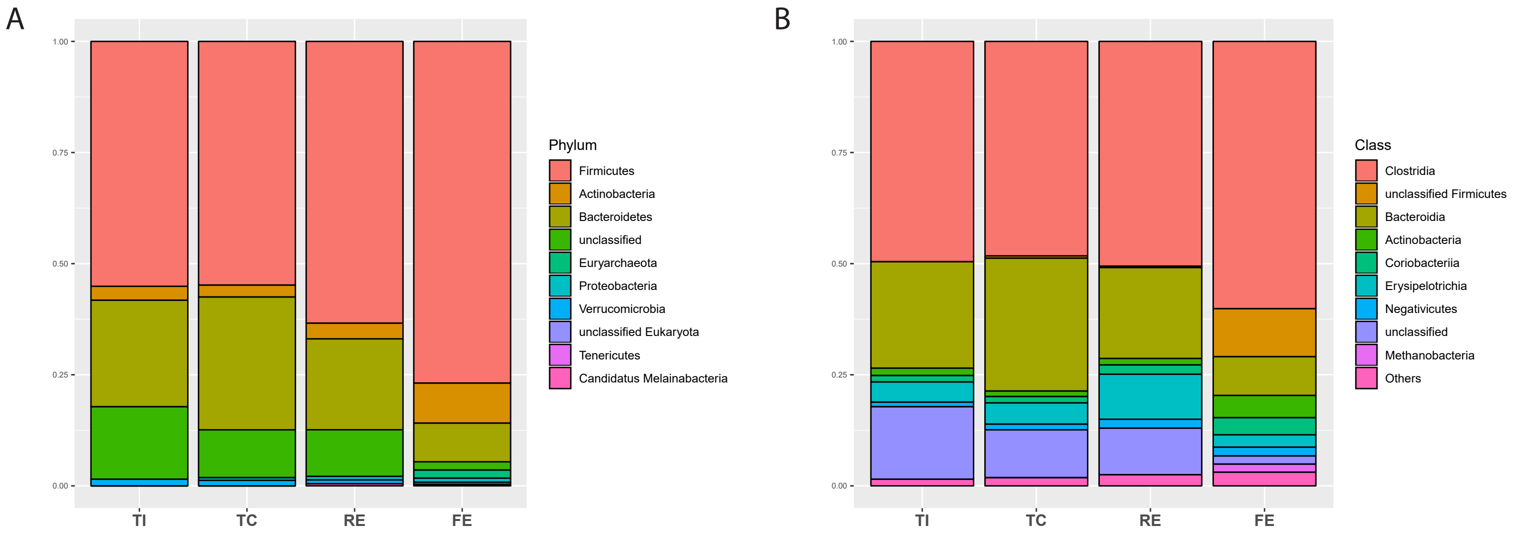

Supplementary Figure 7 - Top 10 most highly abundant phyla (A) and classes (B) in the large intestine; phyla/classes are sorted, in the legends, from the most to the least abundant in the feces samples.

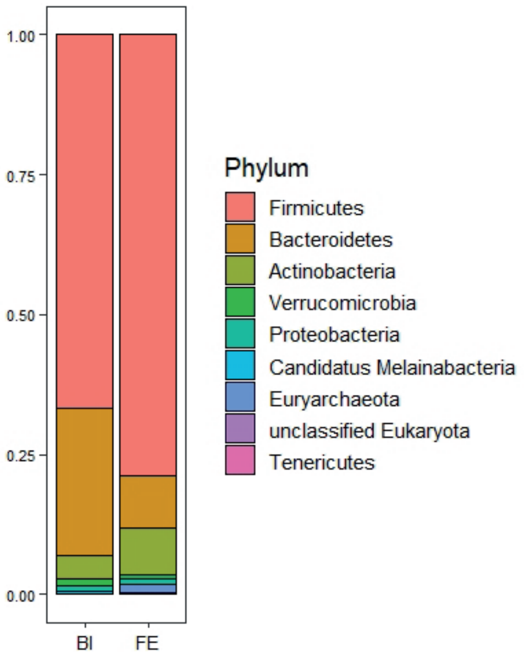

Supplementary Figure 8 - Top 10 most highly abundant phyla in the biopsies dataset, obtained by merging the data from all biopsy locations, and in faeces.

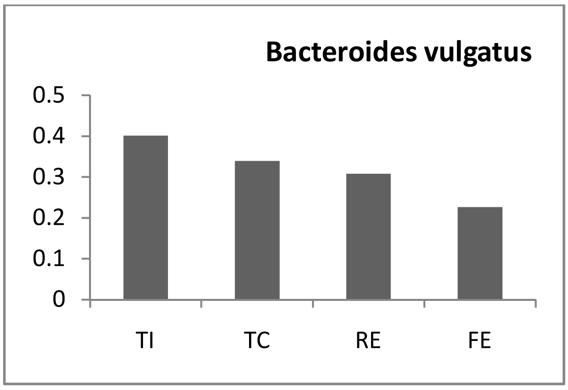
Bacteroides vulgatus
0.5
0.4
0.3
0.2
0.1
0
TI
TC
RE
FE

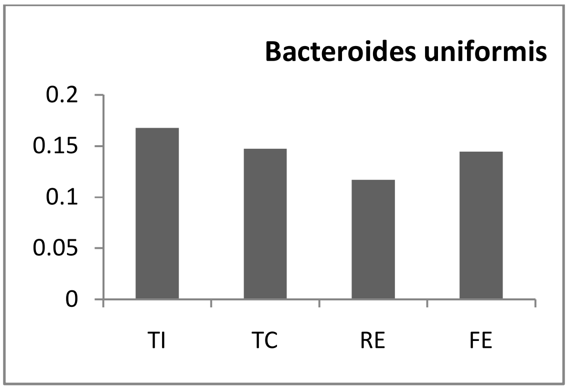
Bacteroides uniformis
0.2
0.15
0.1
0.05
0
TI
TC
RE
FE

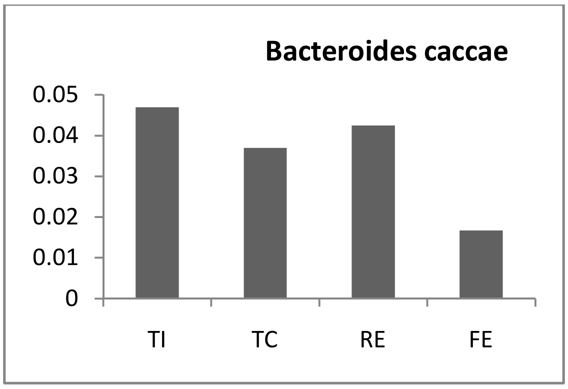
Bacteroides caccae
0.05
0.04
0.03
0.02
0.01
0
TI
TC
RE
FE

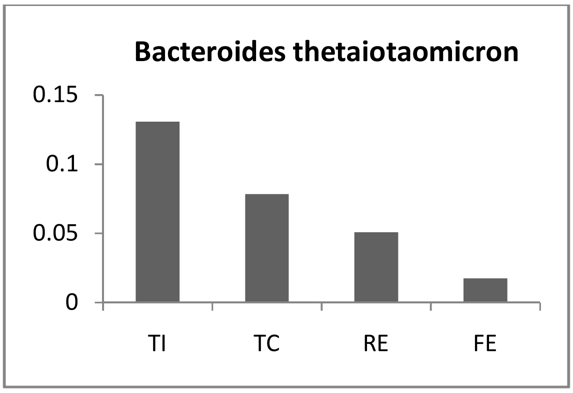
Bacteroides thetaiotaomicron
0.15
0.1
0.05
0
TI
TC
RE
FE

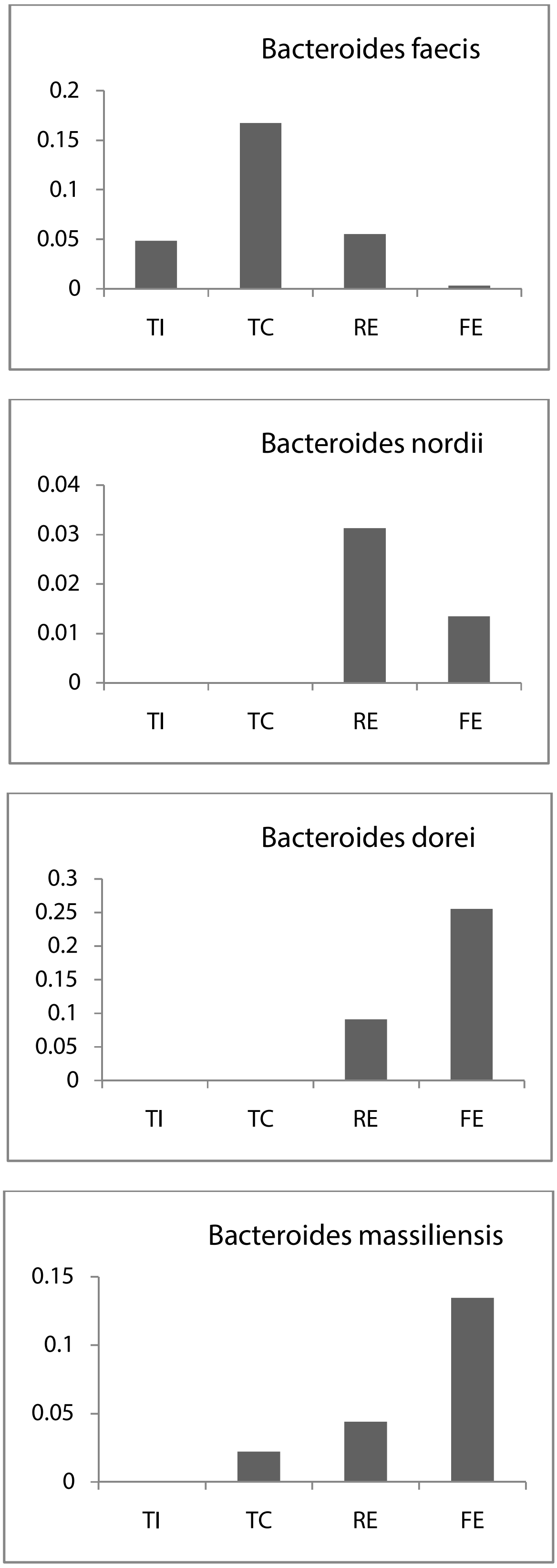

Supplementary figure 9 - Relative abundance bar plots of the main Bacteroides spp. detected in the biopsies samples.

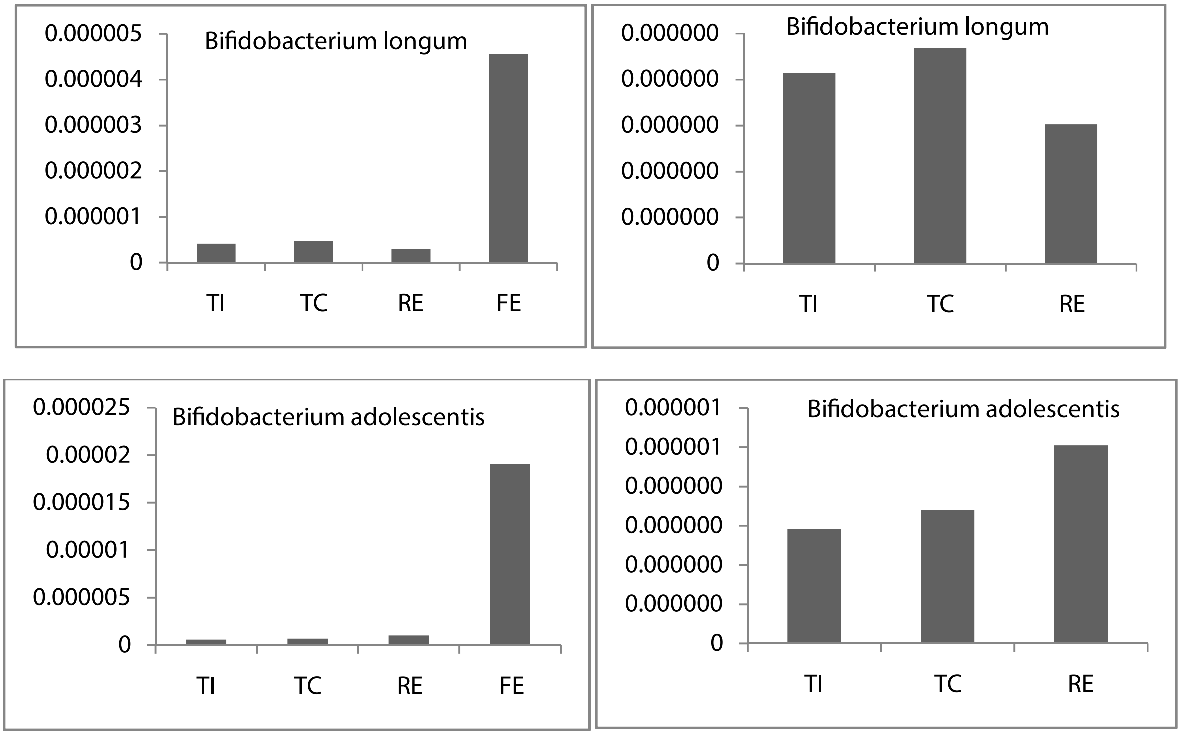

Supplementary figure 10 - Relative abundance bar plots of the main Bifidobacterium spp. detected in the biopsies and faecal samples.

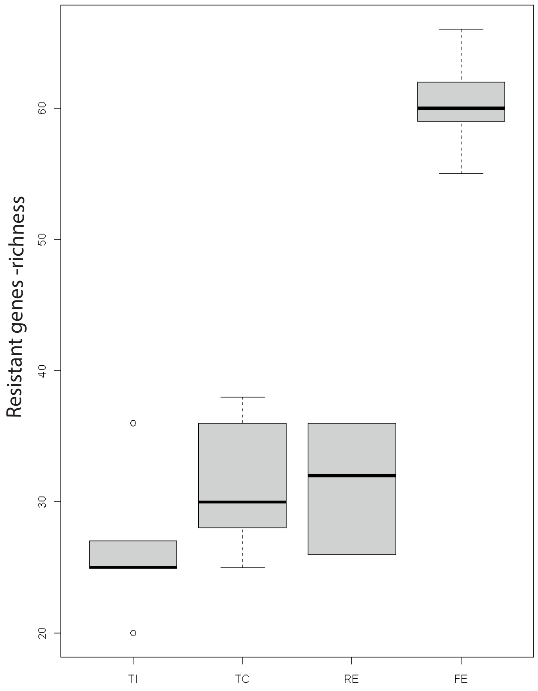

Supplementary figure 11 - Antimicrobial resistant genes richness in all sampling locations. The differences between biopsy locations are not significant (TI vs TC p=0.12; TI vs RE p=0.37).

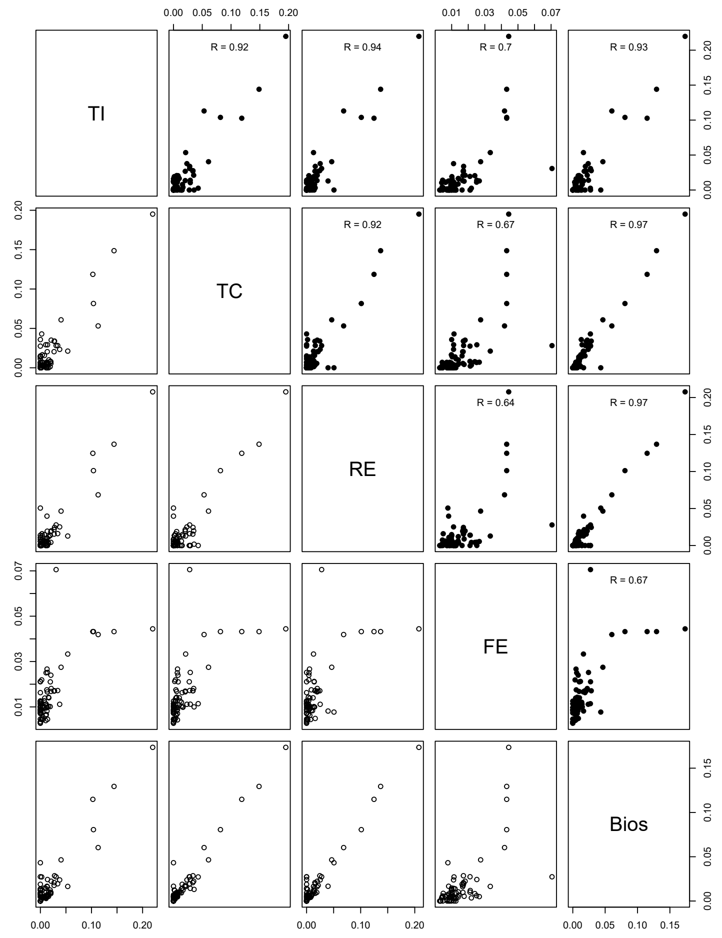

Supplementary figure 12 - Linear correlation between all resistomes.

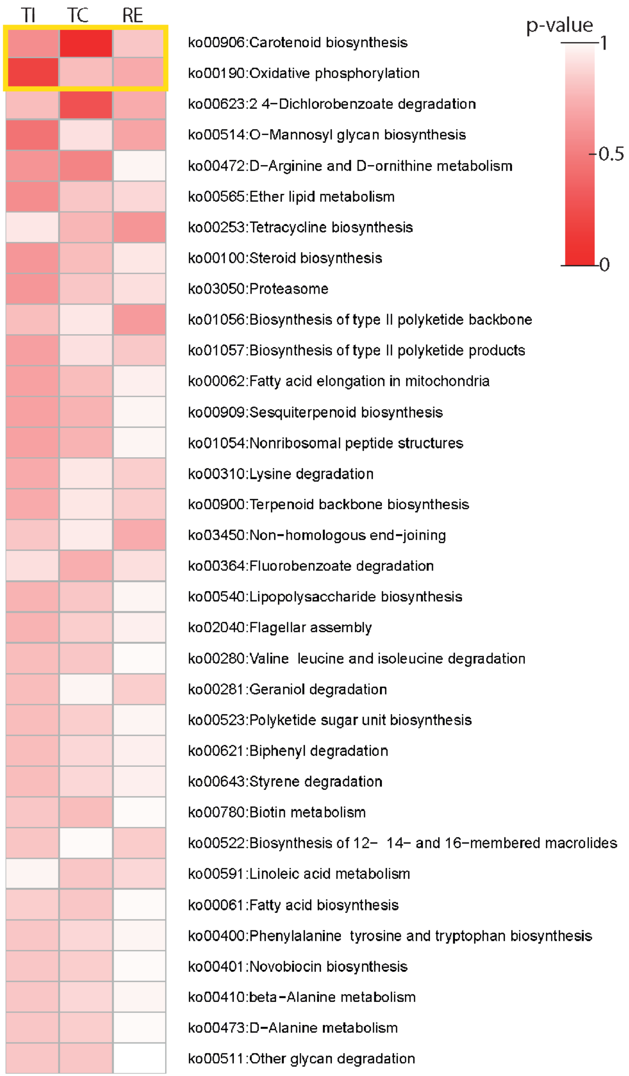

Supplementary Figure 13 - KO orthology by location, results sorted by p-value (Wilcoxon signed rank test). The only two significant pathways are highlighted in yellow.

A

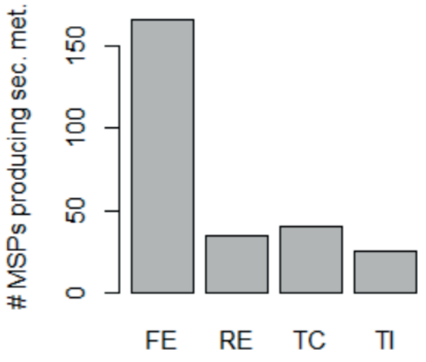

B

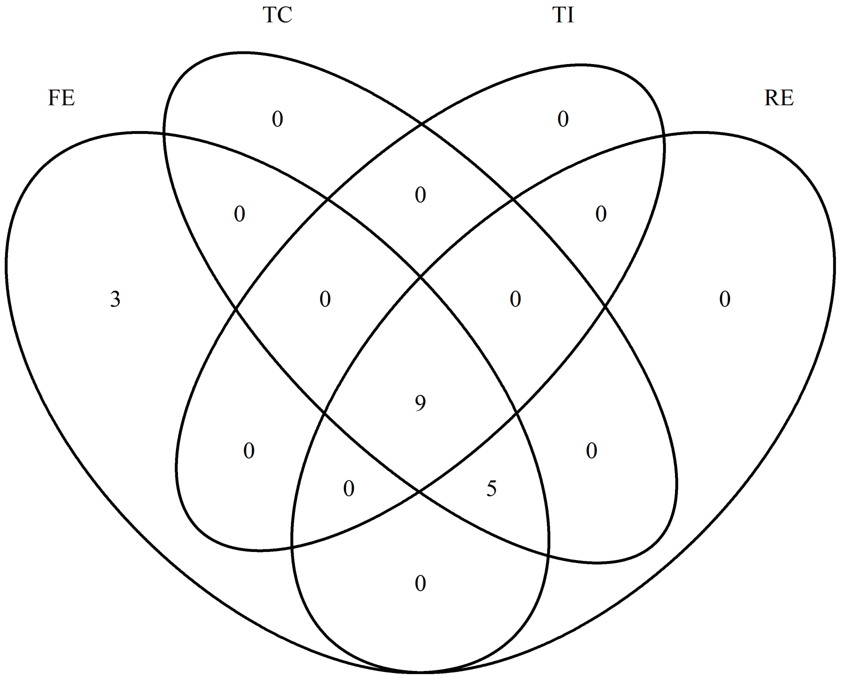

Supplementary Figure 14 - (A) Number of MSPs predicted to produce secondary metabolites in faeces and biopsy-derived samples. (B) Number of predicted secondary metabolites being shared between different samples.

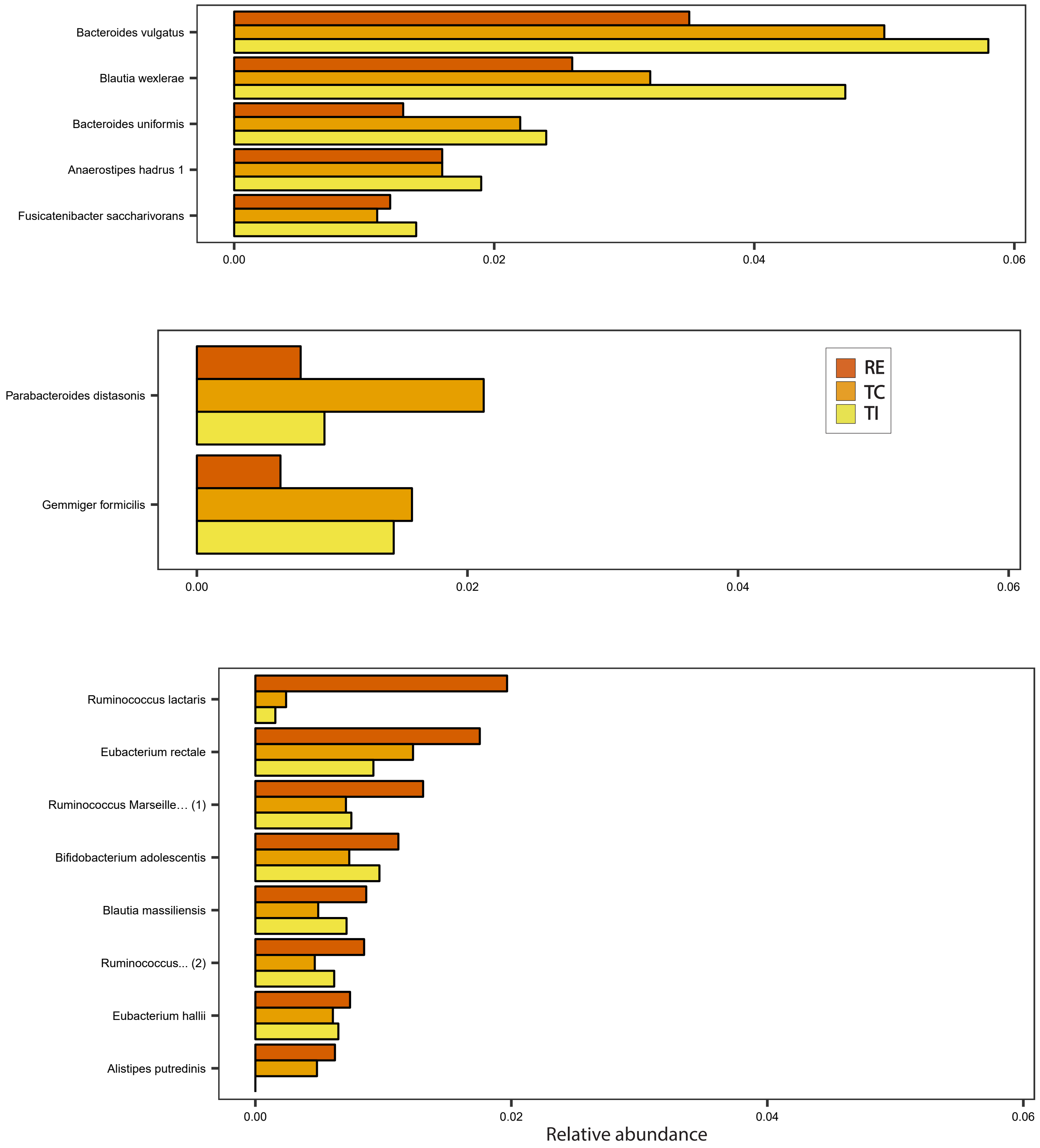

Supplementary figure 15 - Species that were most highly enriched at one biopsy location only, and in at least two patients
